# Testis-Specific Lactate Dehydrogenase (LDH-C_4_) in Skeletal Muscle Enhances a Pika’s Sprint-Running Capacity in Hypoxic Environment

**DOI:** 10.3390/ijerph120809218

**Published:** 2015-08-07

**Authors:** Yang Wang, Lian Wei, Dengbang Wei, Xiao Li, Lina Xu, Linna Wei

**Affiliations:** Research Center for High Altitude Medicine, Qinghai University, Xining 810016, China; E-Mails: yangwangmd@163.com (Y.W.); weilian5318@163.com (L.W.); lixiao2234566@163.com (X.L.); xu020916@163.com (L.X.); weilinnanana@163.com (L.W.)

**Keywords:** testis-specific lactate dehydrogenase C (LDH-C_4_), hypoxia, Qinghai-Tibet Plateau, plateau pika (*Ochotona curzoniae*), skeletal muscle

## Abstract

LDH-C_4_ is a lactate dehydrogenase that catalyzes the conversion of pyruvate to lactate. In mammals, *ldh-c* was originally thought to be expressed only in testis and spermatozoa. Plateau pika (*Ochotona curzoniae*), which belongs to the genus *Ochotona* of the *Ochotonidea* family, is a hypoxia tolerant mammal living 3000–5000 m above sea level on the Qinghai-Tibet Plateau, an environment which is strongly hypoxic. *Ldh-c* is expressed not only in testis and sperm but also in somatic tissues of plateau pika. In this study, the effects of *N*-propyl oxamate and *N*-isopropyl oxamate on LDH isozyme kinetics were compared to screens for a selective inhibitor of LDH-C_4_. To reveal the role and physiological mechanism of LDH-C_4_ in skeletal muscle of plateau pika, we investigated the effect of *N*-isopropyl oxamate on the pika exercise tolerance as well as the physiological mechanism. Our results show that *Ki* of *N*-propyl oxamate and *N*-isopropyl oxamate for LDH-A_4_, LDH-B_4_, and LDH-C_4_ were 0.094 mmol/L and 0.462 mmol/L, 0.119 mmol/L and 0.248 mmol/L, and 0.015 mmol/L and 0.013 mmol/L, respectively. *N*-isopropyl oxamate is a powerful selective inhibitor of plateau pika LDH-C_4_. In our exercise tolerance experiment, groups treated with inhibitors had significantly lower swimming times than the uninhibited control group. The inhibition rates of LDH, LD, and ATP were 37.12%, 66.27%, and 32.42%, respectively. Our results suggested that *ldh-c* is expressed in the skeletal muscle of plateau pika, and at least 32.42% of ATP in the skeletal muscle is catalyzed by LDH-C_4_ by anaerobic glycolysis. This suggests that pika has reduced dependence on oxygen and enhanced adaptation to hypoxic environment due to increased anaerobic glycolysis by LDH-C_4_ in skeletal muscle. LDH-C_4_ in plateau pika plays the crucial role in anaerobic glycolysis and generates ATP rapidly since this is the role of LDH-A_4_ in most species on plain land, which provide evidence that the native humans and animals in Qinghai-Tibet plateau can adapt to the hypoxia environment.

## 1. Introduction

At more than 3000 m above sea level on average, the Qinghai-Tibet Plateau is the highest and largest plateau in the world and possesses a unique environment of nature and geography. Hypoxia and cold environment are the most obvious climate characters on the plateau, which expectedly have profound effects on survival.

Altitude-related health diseases are particularly important in Qinghai-Tibet since more than nearly 12 million settle above 2,200 m on the plateau. Furthermore, every year thousands of people travel from lowland up to the plateau and about 6 million low land immigrants now live there permanently. Tibetans who have been living at high attitude for varying lengths of time, therefore, are considered to be well-adapted to hypoxia conditions and form a unique group on hypoxia physiology. Recently, studies have shown that Tibetans maintain higher arterial oxygen saturation at rest and during exercise with increasing altitude, show reduced loss of aerobic performance [1]. Tibetans have greater hypoxia and hypercapnic ventilatory responsiveness, large lungs, better lung function, and greater lung diffusing capacity than lowlanders [1]. Additional, Tibetans developed only minimal hypoxia pulmonary vasoconstriction [2] and have higher levels of exhaled nitric oxide [3].

Besides, over long-term evolution, many plateau-native animals also have developed their own unique mechanisms to adapt to the harsh plateau environment. They also developed some important characteristics to avoid unfavorable environmental aspects and effectively acquire matter or energy from their environment to ensure their normal growth and reproduction. Plateau pika (*Ochotona curzoniae*), an endemic species of the Qinghai-Tibet Plateau, inhabits meadows at altitudes 3000–5000 m above sea level. The pika is a key species on the Qinghai-Tibetan Plateau, where it plays an important role in biodiversity of the ecosystem [4,5]. Pika fossil samples found on the north edge of the Qinghai-Tibetan plateau are about 37 million years old [6]. During evolution, the pika underwent a series of changes to adapt to the harsh environment and become a high hypoxia-tolerant mammal. First, the pika developed larger pulmonaryalveoli and higher capillary density to obtain oxygen from the hypoxic environment more efficiently [7]. Additionally, pika developed thin walled pulmonary arterioles, blunted hypoxic pulmonary vasoconstriction [8], increased erythrocyte counts [9], reduced mean corpuscular volume [10], changes in hemoglobin (Hb) [11] and 2,3-diphosphoglycerate concentrations [8], and an increased Hb affinity for oxygen [11]. Pikas also have a larger heart and smaller right-to-left ventricular plus septum weights, leading to increased cardiac function [12]. Thirdly, pikas have a high ratio of oxygen utilization by increasing capillary and mitochondrial densities [13] and concentration of myoglobin in tissues [9,12]. In addition to these physiologic mechanisms, pikas have reduced dependence on oxygen by increasing anaerobic glycolysis in skeletal muscle [14] and gluconeogenesis in liver [15]. The molecular basis of these adaptations in the pika have occurred because of a series of genetic evolutionary changes, tissue-specific protein expression, and changes related to altitude, including HIF-1α [16,17], hemoglobin [6], vascular endothelial growth factor (VEGF) [18,19], testis-specific lactate dehydrogenase (LDH-C_4_) [20], pyruvate carboxylase [15], myoglobin [12], cytochromic oxidase [21], inducible nitric oxide synthase (iNOS) [22], and leptin [23,24].

The lactate dehydrogenase (LDH) family enzymes (commission numbers is EC 1.1.1.27.) catalyze the inter-conversion of pyruvate to lactate with the concomitant oxidation/reduction of NADH to NAD^+^ [25]. Different forms of LDH are the product of three different genes: *Ldh-a*, *Ldh-b*, and *Ldh-c* which encode A, B and C subunits, respectively [26,27]. LDH consists of A and B subunits that assemble into homo- or heterotetramers that are distributed in the body in various combinations reflecting the metabolic requirements of different tissues and are consistent with the catalytic properties of the isozymes [28,29]. However, the homotetramer LDH-C_4_ was previously only detected in testis and spermatozoa and not in any other tissues or cells [30,31,32,33]. In our previous study, we identified that *Ldh-c* is expressed not only in testis and sperm but also in somatic tissues of plateau pika [20].

LDH-C_4_ catalyzes the interconversion of pyruvate to lactate with the concomitant oxidation of NADH to NAD^+^, which is essential for the continued production of Adenosine Triphosphate (ATP) by glycolysis [34]. LDH-C_4_ has unique structural and functional properties [35,36,37].Comparative studies of catalytic properties have revealed differences between LDH-C_4_ and other lactate dehydrogenase isozymes [38,39] LDH-C_4_ from different species shows activity against α-keto and α-hydroxy acids of longer carbon chains than those of pyruvate and lactate [35,36,37,40,41,42,43,44,45,46,47]. Substrate specificity differences between LDH isozymes and LDH-C_4_ isozymes from different species suggest that N-substituted oxamates are strong inhibitors of LDH-C_4_ activity [48]. Additional studies investigating the susceptibility of the LDH isozymes to oxamate (a well-known competitive inhibitor of LDH isozymes [49]) and other *N*-substituted oxamates [38,49] led us to synthesize *N*-isopropyl oxamate by attaching the non-polar isopropylic carbon chain to the nitrogen of oxamate. Comparative studies of oxamate and *N*-isopropyl oxamate inhibition of murine LDH isozymes suggest *N*-isopropyl oxamate is a powerful selective inhibitor of mouse LDH-C_4_ [38].

In this paper, we compared inhibition and specificity of *N*-propyl oxamate and *N*-isopropyl oxamate to LDH-A_4_, LDH-B_4_, and LDH-C_4_ of plateau pika. Additionally, we investigated the effect and physiologic mechanism of *N*-isopropyl oxamate on pika exercise tolerance.

## 2. Materials and Methods

### 2.1. Animal Procedures

Plateau pikas were live-trapped in August at Laji Mountain in Xining City, Qinghai Province in China, at altitude of 3,850 m. The total sample size was 40. The temperature was 10–20 °C outside. The average bodyweight of plateau pikas was 198 ± 9 g. All animals used in this study were adults and found to be in good health. The animals were divided into four groups randomly and treated as follows. Group 1 (swimming control group): plateau pikas were injected with 0.5 mL normal saline in each bilateral biceps femoris of hind legs and forced to swim until exhausted. Group 2 (control group): plateau pikas were injected with 0.5 mL normal saline in each bilateral biceps femoris of hind legs. Group 3 (swimming inhibition group): plateau pikas were injected with 0.5 mL 1 mol/L *N*-isopropyl oxamate in each bilateral biceps femoris of hind legs and forced to swim till exhausted. Group 4 (inhibition group): plateau pikas were injected with 0.5 mL 1 mol/L *N*-isopropyl oxamate in each bilateral biceps femoris of hind legs. The exhaust degree of animals were determined by the condition that they were about to sink below the water. Sample size was 10 for all groups above. All animals were forced to rest for 30 min after injection and began the swimming experiment. After experiments, all animals were first anesthetized with sodium pentobarbital (50 mg/kg) and then sacrificed by cervical dislocation immediately before dissection. The cervical arterial blood was collected and stored at 4 °C. The serum was collected from the superstratum of cervical blood followed by centrifugation at 5000 r/min at 4 °C for 10 min. Skeletal muscle was rapidly removed and frozen in liquid nitrogen for long-term storage. All procedures involved in the handling and care of animals were in accordance with the China Practice for the Care and Use of Laboratory Animals and were approved by the China Zoological Society (permit number: GB 14923-2010).

### 2.2. Enzyme and Inhibitor Preparation

The 999 bp, 1005 bp, and 999 bp *Bam*HI*/Xho*I fragment representing the entire *Ldha*, *Ldhb*, and *Ldhc* coding sequence (accession numbers HQ704676, HQ704677, and HQ704678, respectively in GenBank) were amplified by PCR from complementary DNA (cDNA) of plateau pika testis using the Premix Ex Taq Version Kit, respectively (Takara, Japan). The PCR primers for *Ldh-a* were 5'-CGGAATTCATGGCAGCTCTCAAGGATCAG-3' (sense) and 5'-CCGCTCGAGGAACTGCAG CTCCTTCTGGAT-3' (antisense), for *Ldh-b* were 5'-CGGAATTCATGGCAACCCTGAAGGAA AAACTCAT (sense) and 5'-CCGCTCGAGCAGGTCCTTCAGGTCCTTCTGGA-3' (antisense), and for *Ldh-c* were 5’-CGGGATCCATGTCGACAGTCAAGGAGC-3' (sense) and 5'-CCGCTCGAG AAACACCAGGTCCTTCTGGAC-3' (antisense), respectively. PCR conditions were 5 min at 95 °C, 30 cycles of 45 s at 95 °C, 45 s at 65 °C, 1minat 72 °C, and a final elongation step at 72 °C for 10 min. The *Bam*HI*/Xho*I fragment of *Ldh-a* was subsequently cloned into the pCold-SUMO expression vector (BPI), the *Bam*HI*/Xho*I fragments of *Ldh-b* and *Ldh-c* were subsequently cloned into the pET-30a (+) expression vector (Novagen), respectively. The recombinant expression shuttles (pCold-SUMO*-Ldh-a*, pET-30a-*Ldh-b*, and pET-30a-*Ldh-c*) were transformed into *E.coli* (*Escherichiacoli.*) BL21 (DE3) cells.

*E.coli* BL21 (DE3) cells were grown in Lysogeny broth (LB) media and the recombinant protein was expressed in *E.coli* BL21 (DE3) cells. The *E.coli* BL21 (DE3) clone transformed with pCold-SUMO*-Ldh-a*, pET-30a-*Ldh-b*, and pET-30a-*Ldh-c* were inoculated into 1L of LB medium containing 100 μg/mL ampicillin or 50 μg/mL kanamycin and cultured at 37 °C. When the *A*600 reached 0.6, IPTG (isopropyl-β-d-thiogalactopyranoside) was added to a final concentration of 1 mmol/L, followed by further culturing at 25 °C for10 h to induce *Ldh-a*, *Ldh-b*, and *Ldh-c* expression. Bacterial cells were harvested by 4000 r/min centrifugation for 15 min and resuspended in 20 mmol/LPBS (pH 7.0), and disrupted 10 times by repeated freezing and thawing (in liquid nitrogen and 37 °C water bath).Triton X-100 (final concentration of 1%), lysozyme (final concentration of 1%) and DNA enzyme (final concentration of 0.2%) were added during the freeze-thaw cycles. The lysates were centrifuged at 15,000 r/min for 10 min at 4 °C, and the obtained supernatant was used for pika LDH-A_4_, LDH-B_4_, and LDH-C_4_ purification. Purification of the recombinant protein was performed using Ni-NTA resin (QIAGEN). The resins were washed twice with increasing concentrations of imidazole (10 mmol/L, 20 mmol/L, 50 mmol/L, 100 mmol/L, 250 mmol/L, and 500 mmol/L) in phosphate buffered saline (PBS) gradient (50 mmol/L NaH_2_PO_4_, 300 mmol/L NaCl, pH 8.0). Each fraction was collected with individual collection tubes and analyzed by native polyacrylamide gel electrophoresis (PAGE). Elutions with LDH enzyme activity were totally collected. Enzyme liquids were obtained from the elutions after removing imidazoleby ultrafiltration tubes (50 kD, 15 mL, Millipore, USA). An ultimate 150–300 μL amount of LDH isoenzymes were purified from 1 L of bacterial culture with this method. LDH isoenzymes stocks were stored in PBS (pH 8.0) at 4 °C until protein analysis. The purity and activity of LDH-A_4_, LDH-B_4_, and LDH-C_4_ was measured by native PAGE and SDS-PAGE (sodium dodecyl sulfate polyacrylamide gel electrophoresis) as described previously [20].

*N*-isopropyl oxamate and *N*-propyl oxamate were synthetized as previously described [39,50]. Inhibitors were dissolved absolutely in 0.9% normal saline before injecting into the animals. Following experiments, high performance liquid chromatography (HPLC) was used to examine the inhibitor concentration in the blood of the plateau pikas.

### 2.3. Enzyme Kinetics of N-Propyl Oxamate and N-Isopropyl Oxamateon LDH Isozymes

Lactate dehydrogenase activity was determined with ultraviolet (UV) spectrophotometer (Unicon 2800, Shanghai, China) by recording the absorbance change at 340 nm produced by the oxidation of NADH (reduced form of nicotinamide-adenine dinucleotide). Assays were all performed at 37 °C. The *Km* value for pyruvate was calculated from Line weaver-Burk plots. The *Ki* value was determined from *Km* and *Vmax* obtained with or without inhibitor added to the reaction buffer using various concentrations of pyruvate at a constant inhibitor concentration, then plotting the slope (*Km/Vmax*) against inhibitor concentrations. The reagent mixture for the pyruvate to lactate reaction contained 0.15 mmol/L NADH, 50 mmol/L PBS (pH 8.0),and sodium pyruvate gradients as substrate at the following concentrations: 0.1 mmol/L, 0.2 mmol/L, 0.4 mmol/L, 0.6 mmol/L, 0.8 mmol/L for LDH isozymes.Enzymeswere held at a constant inhibitor concentration. The concentration gradients of *N*-propyl oxamate and *N*-isopropyl oxamate were 0.02 mmol/L, 0.04 mmol/L, 0.06 mmol/L, 0.08 mmol/L and 0.1 mmol/L, respectively while substrates, coenzymes, and other buffers were the same as described for their action from pyruvate to lactate outlined above. To provide a △E_340_ of 0.06–0.07 per minute, the enzyme preparation was diluted with phosphate buffer (pH 8.0) when the activity was assayed in a 1 cm light path. The coenzyme was incubated with the buffer used in the assay for 10 min at 37 °C before starting the reaction by adding the substrate. The total reagents added were controlled at 3 mL volumes. All chemicals were analytically pure and purchased from Sangon (Shanghai, China).

### 2.4. Exercise Tolerance Experiments for Plateau Pikas

To measure LDH-C_4_ function in exercise tolerance of plateau pika, forced swimming tests were performed on the animals of Group1 (swimming control group) and Group3 (swimming inhibition group). The swimming pool was a round water channel with 30 cm diameters length and 50 cm deep. Water temperature was controlled at 9–10 °C. Animals were put into the water channel and forced to swim after injection for 30 min. The swimming time of plateau pikas in two groups were recorded by the maximum seconds each animal can survive in the water.

### 2.5. Activity of LDH, the Content of LD and ATP Assay

The samples of skeletal muscle were homogenized on ice as a 1:9 (W/V) dilution in 0.9% physiological saline. The homogenate was centrifuged at 15,000 r/min at 4 °C for 10 min, and the supernatant was collected. The total protein concentration, LDH activity and content of LD (lactic acid) were determined using commercially available assay kits according to the manufacturer’s instructions (Nanjing Jiancheng Bioengineering Institute, China). The amount of ATP was measured by the luciferin-luciferase method [51] following the protocol of ATP detection kit (Beyotime, China). The luminescence from a 100 μL sample was assayed in a luminometer (Promega, GloMax20/20, USA) together with 100 μL ATP detection buffer from the ATP detection kit. Standard curves were also generated and the protein concentration of each sample was determined using the BCA Protein assay (Pierce, USA).

### 2.6. Data Analysis

All values were expressed as mean ± standard deviation (SD). Statistical analysis was performed by one-way analysis of variance (ANOVA) and Duncan’s test using SPSS 22.0 (SPSS Inc., Chicago, IL, USA). A value of *p* < 0.01 was considered very significant. A value of *p* < 0.05 was considered statistically significant.

## 3. Results

### 3.1. Enzyme Kinetic Properties of N-Propyl Oxamate and N-Isopropyl Oxamate on LDH Isozymes

**Figure 1:**
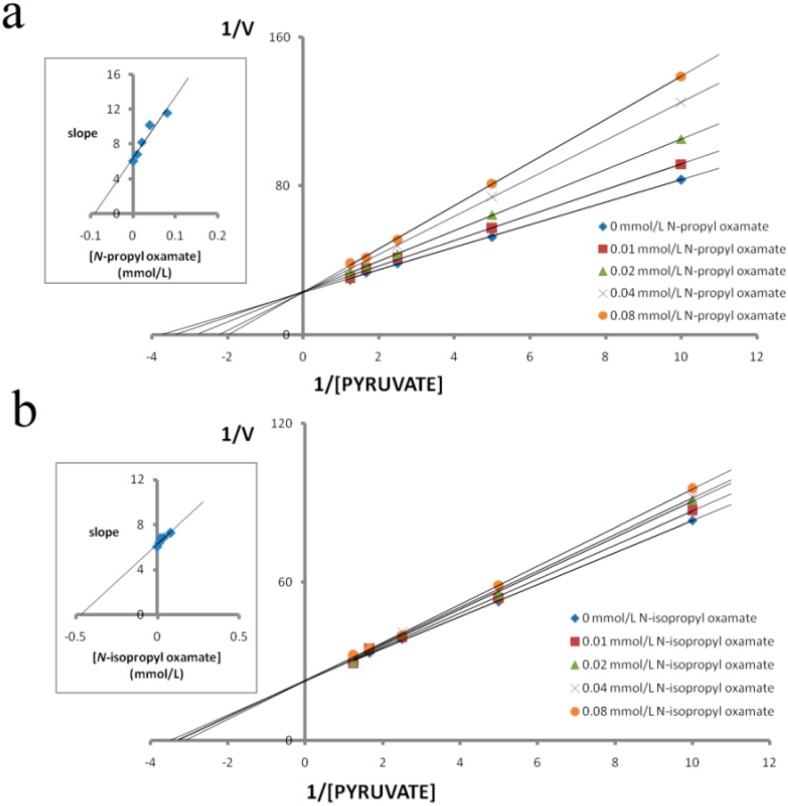
Effect of pyruvate on the inhibitory activity of *N-*propyloxamate and *N-*isopropyl oxamateon plateau pika LDH-A_4_. Reciprocal values of *V* were calculated taking the reciprocal values of Δ340 nm/min, plots of reciprocal reaction velocity *versus* reciprocal pyruvate concentration at a constant NADH concentration. The concentrations of pyruvate used were 0.1 mmol/L, 0.2 mmol/L, 0.4 mmol/L, 0.6 mmol/L and 0.8 mmol/L. NADH concentration was kept at 0.15 mmol/L. The *Km* of LDH-A_4_ for pyruvate was 0.260 mmol/L. (◆) assays with 0 mmol/L, (■) 0.01 mmol/L, (▲) 0.02 mmol/L, (×) 0.04 mmol/L and (●) 0.08 mmol/L of *N-*propyl oxamate or *N-*isopropyl oxamate. Upper left: determination of *Ki* from replot of slope values against inhibitor concentrations. The *Ki* of *N-*propyl oxamate and *N-*isopropyl oxamate for LDH-A_4_ were 0.094 mmol/L and 0.462 mmol/L, respectively.

**Figure 2:**
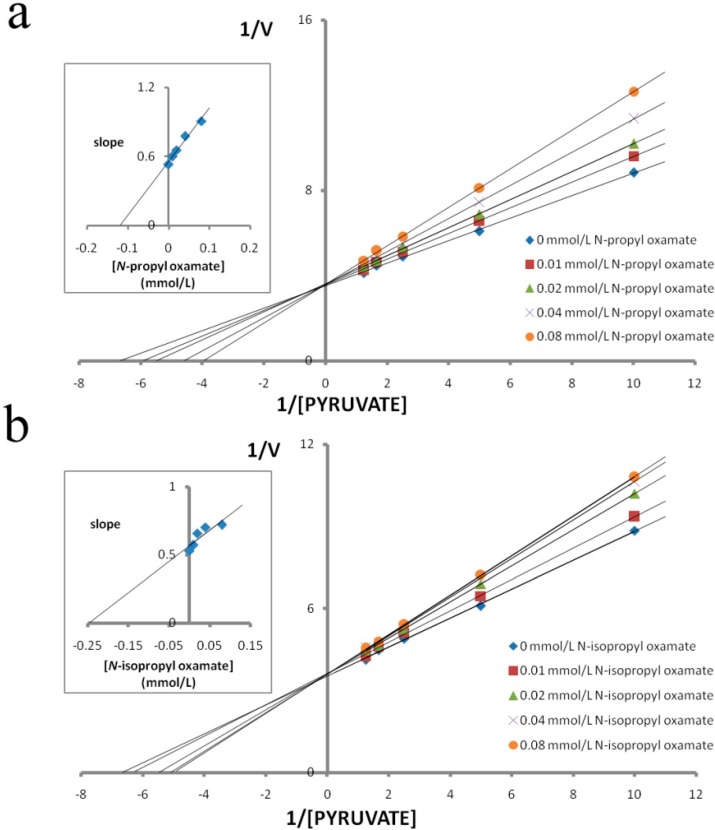
Effect of pyruvate on the inhibitory activity of *N-*propyl oxamate and *N-*isopropyl oxamate on plateau pika LDH-B_4_. Reciprocal values of *V* were calculated taking the reciprocal values of Δ340 nm/min, plots of reciprocal reaction velocity *versus* reciprocal pyruvate concentration at a constant NADH concentration. The concentrations of pyruvate used were 0.1 mmol/L, 0.2 mmol/L, 0.4 mmol/L, 0.6 mmol/L, and 0.8 mmol/L. NADH concentration was kept at 0.15 mmol/L. The *Km* of LDH-B_4_for pyruvate was 0.172 mmol/L. (◆) assays with 0 mmol/L, (■) 0.01 mmol/L, (▲) 0.02 mmol/L, (×) 0.04 mmol/L and (●) 0.08 mmol/L of *N*-propyl oxamate or *N-*isopropyl oxamate. Upper left: determination of *Ki* from replot of slope values against inhibitor concentrations. The *Ki* of *N-*propyl oxamate and *N-*isopropyl oxamate for LDH-B**_4_** were 0.119 mmol/L and 0.248 mmol/L, respectively.

**Figure 3:**
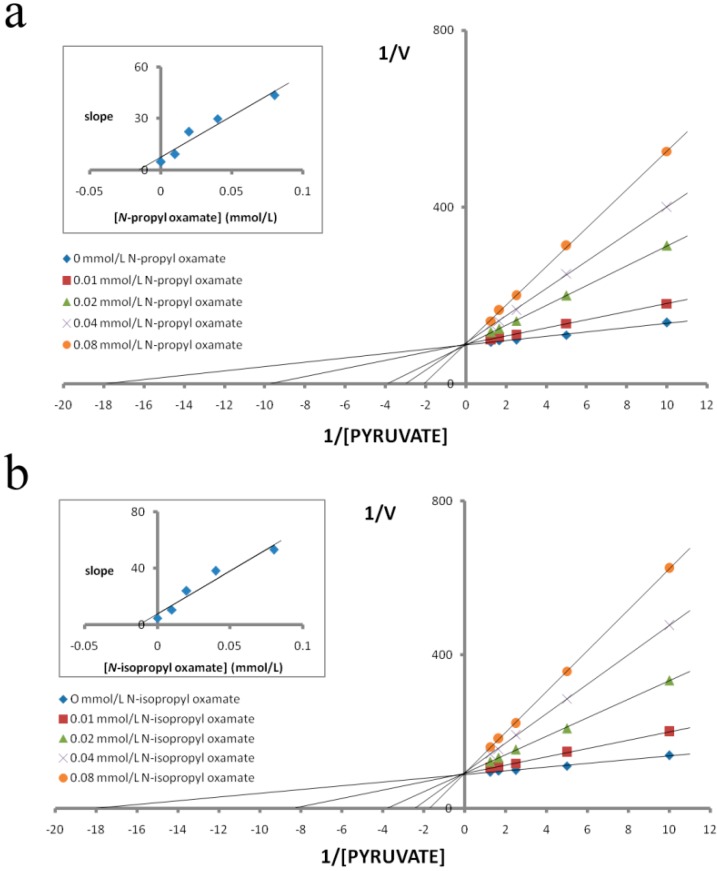
Effect of pyruvate on the inhibitory activity of *N-*propyl oxamate and *N-*isopropyl oxamateon plateau pika LDH-C_4_. Reciprocal values of *V* were calculated taking the reciprocal values of Δ340 nm/min, plots of reciprocal reaction velocity *versus* reciprocal pyruvate concentration at a constant NADH concentration. The concentrations of pyruvate used were 0.1 mmol/L, 0.2 mmol/L, 0.4 mmol/L, 0.6 mmol/L, and 0.8 mmol/L. NADH concentration was kept at 0.15 mmol/L. The *Km* of LDH-C_4_ for pyruvate was 0.052 mmol/L. (◆)assays with 0 mmol/L, (■) 0.01 mmol/L, (▲) 0.02 mmol/L, (×) 0.04 mmol/L and (●) 0.08 mmol/L of (a) *N*-propyl oxamate or (b) *N-*isopropyl oxamate. Upper left: determination of *Ki* from replot of slope values against inhibitor concentrations. The *Ki* of *N-*propyl oxamate and *N-*isopropyl oxamate for LDH-C_4_ were 0.015 mmol/L and 0.013 mmol/L, respectively.

**Figure 4:**
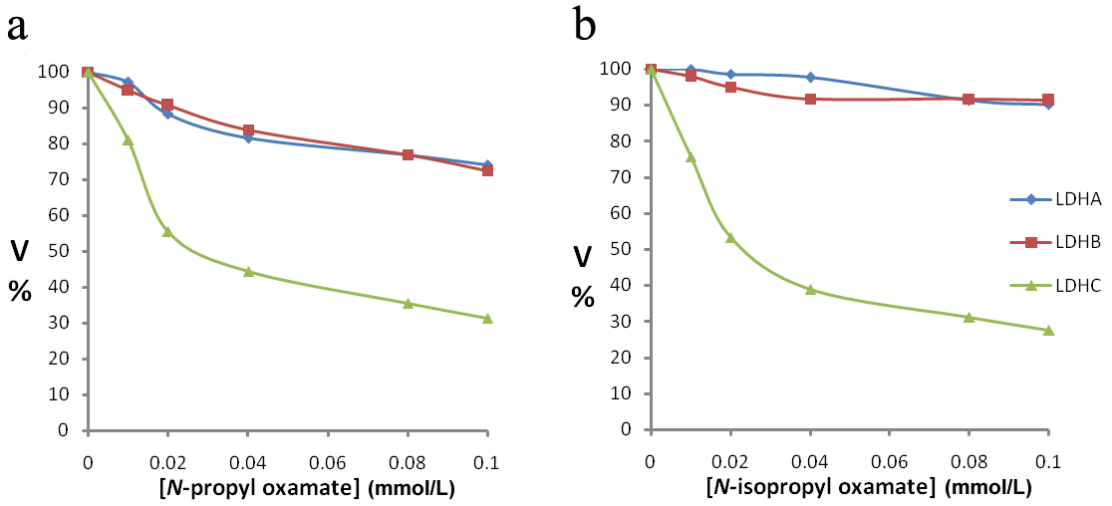
(**a**) Effect of increasing concentrations of *N-*propyloxamate and *N-*isopropyl oxamate on plateau pika LDH isozymes. *N-*propyl oxamate. (**b**) *N-*isopropyl oxamate. Pyruvate was used as substrate. Velocities were calculated taking the maximum activity without the *N*-propyl oxamate or *N*-isopropyl oxamateas 100%. *N-*propyl oxamate or *N-*isopropyl oxamate were added at the concentration of 0.01 mmol/L, 0.02 mmol/L, 0.04 mmol/L, 0.08 mmol/L and 0.1 mmol/L. (◆) LDH-A_4_, (■) LDH-B_4_, (▲) LDH-C_4_.

We purified LDH isozymes from *E. coli* and characterized how *N*-propyl oxamate and *N-*propyl oxamate affected enzyme kinetics, as shown in Figure 1, Figure 2 and Figure 3. *Ki* of LDH-A_4_ by *N*-propyl oxamate and *N*-isopropyl oxamate was 0.094 mmol/L and 0.462 mmol/L, respectively, shown in Figure 1; *Ki* of LDH-B_4_ by *N*-propyl oxamate and *N*-isopropyl oxamate was 0.119 mmol/L and 0.248 mmol/L, respectively, shown in Figure 2; *Ki* of LDH-C_4_ by *N*-propyl oxamate and *N*-isopropyl oxamate was 0.015 mmol/L and 0.013 mmol/L, respectively, shown in Figure 3. Our results indicate the LDH-A_4_ and LDH-B_4_ inhibition by *N*-propyl oxamate is five and two times higher than that of *N*-isopropyl oxamate, but the *Ki* of LDH-C_4_ by the two inhibitors was almost the same. The above results suggest the two inhibitors have different inhibition effects on LDH-A_4_ and LDH-B_4_, but were comparable for LDH-C_4_. *N*-isopropyl oxamate was more specific for LDH-C_4_ than *N*-propyl oxamate at concentrations up to 0.1 mmol/L, as shown in Figure 4. When the concentration of *N*-isopropyl oxamate was 0.1 mmol/L, LDH-C_4_ was inhibited by 70% while LDH-A_4_and LDH-B_4_ were only inhibited by less than 10%. Therefore, *N*-isopropyl oxamate was selected as the optimal inhibitor to study the function of LDH-C_4_ in the exercise tolerance of plateau pikas in the further experiments.

### 3.2. Swimming Time and Biochemistry Assay after Exercise Tolerance Experiment

**Figure 5:**
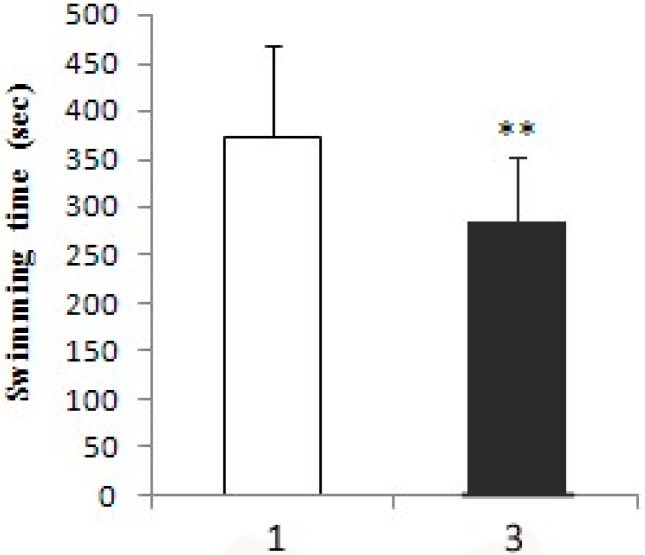
Swimming time of treated and untreated pikas. Group 1 pikas were injected with 0.5 mL of normal saline in each biceps femoris and swam until exhausted. Group 3 pikas were injected with 0.5 mL 1mol/L isopropyl oxamate in the biceps femoris of hind legs and swam until exhausted. All animals were forced to rest for 30 min after injection. Water temperature: 9–10 °C. All data were expressed as mean ± SD; the sample size was 10 for each group. ** *p* < 0.01. The average swimming time of Group 3 (284 ± 93 s) is significantly lower than that of Group 1 (374 ± 67 s).

**Figure 6:**
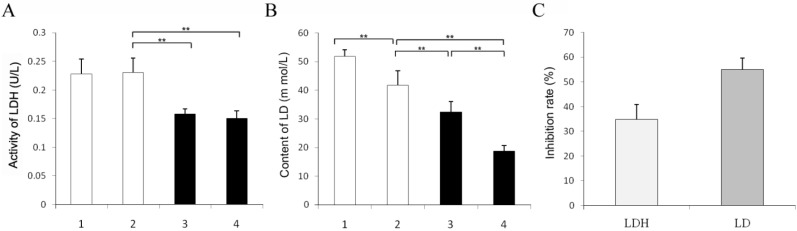
Effects of *N*-isopropyl oxamate on the activity of LDH, content of LD and ATP in the serum of plateau pika. (**A**) LDH activity in the serum of plateau pika with different treatments. (**B**) LD content in the serum of plateau pika with different treatments. (**C**) Inhibition rates of LDH and LD in the serum. Group 1: taking samples when swimming till exhausted with each bilateral biceps femoris of hind legs injected 0.5 mL normal saline; Group 2: taking samples with each bilateral biceps femoris of hind legs injected 0.5 mL normal saline; Group 3: taking samples when swimming till exhausted with each bilateral biceps femoris of hind legs injected 0.5 mL 1mol/L isopropyl oxamate; Group 4: taking samples with each bilateral biceps femoris of hind legs injected 0.5 mL 1 mol/L isopropyl oxamate. All animals were forced to rest for 30 min after injection. Water temperature: 9–10 °C. All data were expressed as mean ± SD; sample size was 10 for each group. The activity of LDH in Group 1–4 were 0.23 ± 0.026 U/mg, 0.23 ± 0.025 U/mg and 0.16 ± 0.009 U/mg and 0.15 ± 0.014 U/mg, respectively; the LD content in Group 1–4 were 51.86 ± 2.23 mmol/g, 41.71 ± 5.04 mmol/g and 32.29 ± 3.76 mmol/g and 18.80 ± 1.97 mmol/g, respectively; the inhibition rates of LDH and LD in the serum were 34.87% and 54.93 %, respectively. ** *p* < 0.01.

**Figure 7:**
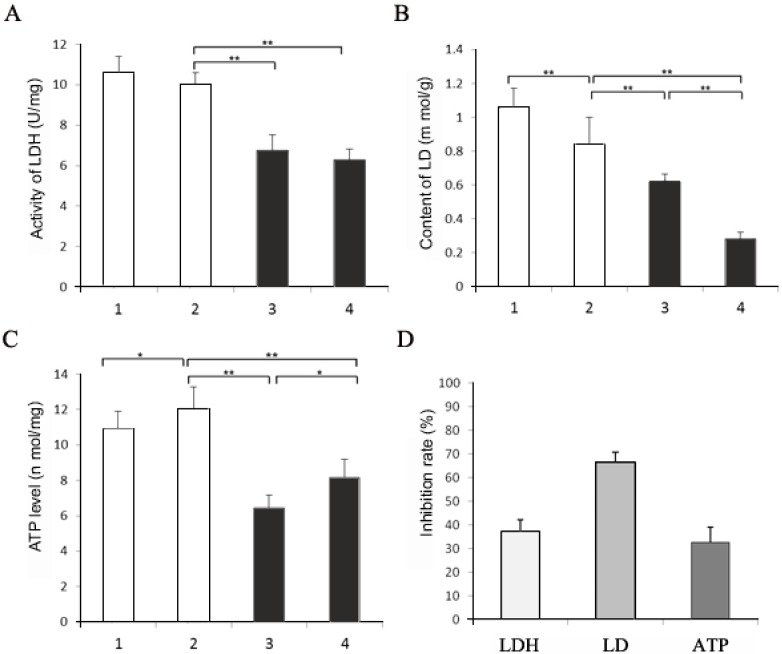
Comparison of LD, LDH, ATP and their inhibition rate of plateau pika skeletal muscle with different treatments. (**A**) LDH activity in the skeletal muscle of plateau pika. (**B**) LD content in the skeletal muscle of plateau pika. (**C**) ATP level in the skeletal muscle of plateau pika. (**D**) Inhibition rates of LDH, LD and ATP in the skeletal muscle. Group 1: taking samples when swimming till exhausted with each bilateral biceps femoris of hind legs injected 0.5 mL normal saline; Group 2: taking samples with each bilateral biceps femoris of hind legs injected 0.5 mL normal saline; Group 3: taking samples when swimming till exhausted with each bilateral biceps femoris of hind legs injected 0.5 mL 1mol/L isopropyl oxamate; Group 4: taking samples with each bilateral biceps femoris of hind legs injected 0.5 mL 1 mol/L isopropyl oxamate. All animals were forced to rest for 30 min after injection. Water temperature: 9–10 °C. All data were expressed as mean ± SD; sample size was 10 for each group. The activity of LDH in Group 1–4 were 10.59 ± 0.79 U/mg, 10.01 ± 0.59 U/mg, 6.74 ± 0.77 U/mg and 6.30 ± 0.50 U/mg, respectively; the LD content in Group 1–4 were 1.06 ± 0.11 mmol/g, 0.84 ± 0.16 mmol/g, 0.62 ± 0.043 mmol/g and 0.28 ± 0.039 mmol/g, respectively; the ATP level in Group 1–4 were 10.96 ± 0.94 nmol/mg, 12.06 ± 1.23 nmol/mg, 6.43 ± 0.75 nmol/mg and 8.15 ± 1.03 nmol/mg, respectively; the inhibition rates of LDH, LD and ATP in the skeletal muscle were 37.12%, 66.27%, and 32.42%, respectively. * *p* < 0.05, ** *p* < 0.01.

When pikas were injected with 1 mL of 1 mol/L *N*-isopropyl oxamate in hind legs for 30 min, HPLC showed the inhibitor concentration was 0.08 mmol/L in the blood. As shown in Figure 4, at this concentration, LDH-C_4_ was inhibited by 70% while LDH-A_4_ and LDH-B_4_was only slightly inhibited. Decline of exercise tolerance was indicated by decreased swimming time and reduced LDH, LD and ATP in skeletal muscle. Figure 5 describes the average swimming time of Group 1 and Group 3, which was 374 ± 67 s and 284 ± 93 s, respectively. The swimming time of swimming inhibition group pikas was significantly lower than that of swimming control group (*p* < 0.01). In addition, as shown in Figure 6 and Figure 7, LDH, LD and ATP of the LDH-C_4_ inhibited group (Group 3 and 4) in skeletal muscle and serum of plateau pikas were also lower than that of non-inhibited group (Group 1 and 2) (*p* < 0.05, *p* < 0.01). LDH and LD inhibition rates by *N*-isopropyl oxamate in the serum were 34.87% and 54.93%, respectively; and those in the skeletal muscle were 37.12%, 66.27% and 32.42% for LDH, LD and ATP, respectively. LD was higher after swimming while ATP was lower. LDH levels were relatively stable throughout the experiment, suggesting that exercise generates lactate and consumes ATP, but does not affect the LDH activity. Together, our results suggest *N*-isopropyl oxamate down-regulates the activity of LDH-C_4_, likely influencing energy metabolism and leads to declined exercise tolerance of plateau pikas.

## 4. Discussion

In our previous study, we showed that *Ldh-c* is expressed in somatic tissues of plateau pika [20]. LDH-C_4_ is a member of the lactate dehydrogenase family of isoenzymes that catalyze the terminal reaction in the glycolytic pathway [52,53]. LDH-C_4_ displays a unique structure and functional properties [52,53].

The enzymatic kinetic properties of LDH-C_4_ have been studied in detail in other species. The biochemical properties differentiating LDH-C_4_ from the other LDH isoforms may contribute to the high glycolytic flux. Compared with LDH-A_4_, LDH-C_4_ has a low *Km* for pyruvate (~0.030 mmol/L) and a high *Km* for lactate (~2.0 mmol/L) [35,38,54,55]. This finding implies that LDH-C_4_ has an affinity for pyruvate that is 60-fold higher than that for lactate and suggests that pyruvate turnover to lactate may be high even at high concentrations of endogenous or extracellular lactate [35,38,54,55]. This theory is supported by experiments in which addition of excess lactate (50-fold excess in relation to pyruvate) did not influence ATP production in capacitating spermatozoa [56]. We found that the enzyme activity of the pika LDH-C_4_ was significantly higher and less sensitive to lactate inhibition than that of LDH-A_4_ and LDH-B_4_ (*p* < 0.01). Compared with LDH-A_4_ and LDH-B_4_, LDH-C_4_ had a low *Km* for pyruvate (~0.052 mmol/L) and a high *Km* for lactate (~4.934 mmol/L); and the affinity of LDH-C_4_ for pyruvate is 90-fold higher than that for lactate. These results suggest that the biochemical properties separating LDH-C_4_ from the other LDH isoforms may contribute to the high glycolytic flux.

Previous studies demonstrated that that disruption of *Ldh-c* or inhibition of LDH-C_4_ in sperm led to rapid decline in sperm ATP levels [34,57], a decrease in progressive motility, and a failure to develop hyperactivated motility. Metabolic tracing experiments revealed that all consumed^13^ C labeled pyruvate added in sperm culture medium was converted to lactate rather than oxidized in the tricarboxylic acid cycle. The ATP concentration was increased by more than 50% in the presence of exogenous pyruvate [56]. When carbonyl cyanide mchlorophenylhydrazone (CCCP) and NaCN was applied to suppress the oxidative phosphorylation in mitochondria, the vigorous motility of sperm was maintained and the amount of ATP was kept at the equivalent level to that without CCCP [56,58]. These results suggest that LDH-C_4_ is the key factor of sperm glycolysis, which has an important role in providing the ATP required for sperm motility [34,58,59]. Pikas are sprint-running animals. Given the results in this study, we propose that the expression of *Ldh-c* in skeletal muscle of plateau pikas enhance their anaerobic glycolysis and reduce dependence on oxygenin hypoxic environments.

Studies found that the α-keto and α-hydroxy dicarboxylic acids with a polar group at the end of the carbon chain were not utilized by murine LDH-C_4_ [35,57,38,60], but murine LDH-C_4_ did have high affinity for α-ketoisocaproate containing non-polar carbon side chains [38,48]. The increase in effectiveness of substrates and inhibitors induced by non-polar substituents can only be due to hydrophobic bonding in the enzyme–inhibitor or enzyme–substrate complex [49,60]. The comparative study of oxamate and *N-*isopropanyl oxamate’s inhibition on murine LDH isozymes showed the isopropylic carbon chain conferred a high affinity for LDH-C_4_, which had the highest LDH-C_4_ inhibition (*K_i_ =* 0.014 mmol/L). The lowest inhibition occurred with LDH-1 (*K*_i_ *=* 0.400 mmol/L) and LDH-5 (*K*_i_ *=* 0.800 mmol/L) [38]. In addition, the glycine, threonine and leucine content of murine LDH-C_4_ was greater than in the other isozymes [61]. Leucine, containing a non-polar R group formed by a branched carbon chain, is exceptionally high in mouse LDH-C_4_, which contains 40 more residues of leucine than LDH-A_4_ and LDH-B_4_ [61]. According to the amino acid constitution of plateau pika LDH-C subunit, we found that the pika LDH-C_4_ contains 26 and 20 more residues of isoleucine than LDH-A_4_ and LDH-B_4_. Likely, leucine or isoleucine residues in pika LDH-C_4_ form part of the hydrophobic region present only at the active site of murine LDH-C_4_ [38] or plateau pika LDH-C_4_. To find a powerful selective inhibitor of plateau pikaLDH-C_4_, we compared the inhibition and specificity of *N*-propyl oxamate and *N*-isopropyl oxamate to LDH-A_4_, LDH-B_4,_ and LDH-C_4_ of plateau pika. Our results show that the *Ki* of *N-*propyl oxamate and *N-*isopropyl oxamate for LDH-A_4_, LDH-B_4_ and LDH-C_4_ were 0.094 mmol/L and 0.462 mmol/L, 0.119 mmol/L and 0.248 mmol/L, and 0.015 mmol/L and 0.013 mmol/L, respectively. LDH-C_4_ was inhibited up to 70% at 0.1 mmol/L concentration of *N*-propyl oxamate or *N*-isopropyl oxamate. However, LDH-A_4_ and LDH-B_4_ was inhibited 20% by *N*-propyl oxamate, and their enzymatic activities were hardly affected by *N*-isopropyl oxamate. These results indicated that *N*-propyl oxamate had a higher affinity and inhibitory effect on plateau pika LDH-A_4_, LDH-B_4_, and LDH-C_4_, but *N*-isopropyl oxamate had higher inhibition and specificity for LDH-C_4_ compared with *N*-propyl oxamate. The affinity difference of *N*-propyl oxamate and *N*-isopropyl oxamate for LDH-C_4_ is probably due to the hydrophobicity difference between the propyl group of *N*-propyl oxamate and isopropyl group of *N*-isopropyl oxamate [38,49]. Therefore, the *N*-isopropyl oxamate is a powerful selective inhibitor of plateau pika LDH-C_4_.

To reveal the role and physiological mechanism of LDH-C_4_ in skeletal muscle of plateau pika, we investigated the effect of *N*-isopropyl oxamate on the pika exercise tolerance as well as the physiological mechanism. Our results indicated that injection of 1 mol/L *N*-isopropyloxamate in the biceps femoris of plateau pika for 30 min resulted in blood concentration of *N*-isopropyl oxamate of 0.08 mmol/L. Swimming times of the treated pikas decreased significantly compared untreated pikas. LDH activity and LD content in serum of treated pikas decreased significantly compared to untreated animals, and the inhibition of LDH and LD by *N*-isopropyl oxamate was 34.87% and 54.93%, respectively. Similarly, LDH activity and LD and ATP content in biceps femoris of treated pikas decreased significantly compared to untreated animals, and inhibition of LDH, LD, and ATP by *N*-isopropyl oxamate was 37.12%, 66.27%, and 32.42%, respectively. We found that enzyme activity of LDH-C_4_ is almost five times and three times more than that of LDH-A and LDH-B, the distribution of six LDH isoenzymes (B4, AB3, A2B2, A3B1, A4 and C4) in pika skeletal muscle had been observed [20]. Since LDHA is the main isozyme subunit in the skeletal muscle, LDH-C_4_ should account for 40–50% of total LDH enzyme activity. The inhibition of the LDH-C_4_ effect resulted in the change of biochemical index. *N-*isopropyl oxamate inhibited LDH-C_4_ 70% without affecting the enzymatic activity of the other two isozymes*in vitro* [38]. Similar results were observed with plateau pika LDH-A_4_, LDH-B_4_, and LDH-C_4_ inhibition caused by *N-*isopropyl oxamate in this research.

## 5. Conclusions

Collectively, our results suggest that decreased ATP in skeletal muscle leads to a decline in the sprint-running tolerance of plateau pika in hypoxic environment due to inhibition of LDH-C_4_ enzymatic activity. Pika have reduced oxygen dependence and enhanced adaptation to hypoxic environments due to increased anaerobic glycolysis by LDH-C_4_ in skeletal muscle. LDH-C_4_ in plateau pika may play the crucial role in anaerobic glycolysis and generate ATP rapidly since this is the role of LDH-A_4_ in most species on plain land.

To provide a basis for the diagnosis, prevention and treatment of acute and chronic plateau disease in Qinghai-Tibet plateau, which is low oxygen, strong radiation and cold, this research can help reveal the physiology and biochemistry mechanism as well as adaption and acclimatization of native animals and residents.
